# Interfacial Hydrogen Bonds and Their Influence Mechanism on Increasing the Thermal Stability of Nano-SiO_2_-Modified Meta-Aramid Fibres

**DOI:** 10.3390/polym9100504

**Published:** 2017-10-12

**Authors:** Chao Tang, Xu Li, Zhiwei Li, Jian Hao

**Affiliations:** 1 College of Engineering and Technology, Southwest University, Chongqing 400715, China; lixuqq@email.swu.edu.cn (X.L.); lizhiwei5001@163.com (Z.L.); 2 School of Electronics and Computer Science, University of Southampton, Southampton SO171BJ, UK; 3 Laboratory of Power Transmission Equipment & System Security and New Technology, Chongqing University, Chongqing 400044, China; haojian2016@cqu.edu.cn

**Keywords:** Nano SiO_2_, meta-aramid fibre, interfacial hydrogen bonds, doping, microscopic mechanism

## Abstract

For further analysis of the effect of nano-doping on the properties of high polymers and research into the mechanism behind modified interfacial hydrogen bonds, a study on the formation probability of nano-SiO_2_/meta-aramid fibre interfacial hydrogen bonds and the strengthening mechanism behind interfacial hydrogen bonds on the thermal stability of meta-aramid fibres using molecular dynamics is performed in this paper. First, the pure meta-aramid fibre and nano-SiO_2_/meta-aramid fibre mixed models with nanoparticle radiuses of 3, 5, 7 and 9 Å (1 Å = 10^−1^ nm) are built, and then the optimization process and dynamics simulation of the models are conducted. The dynamics simulation results indicate that the number of hydrogen bonds increase due to the doping by nano-SiO_2_ and that the number of interfacial hydrogen bonds increases with the nanoparticle radius. By analysing the hydrogen bond formation probability of all the atom pairs in the mixed model with pair correlation functions (PCFs), it can be observed that the hydrogen bond formation probability between the oxygen atom and hydrogen atom on the nanoparticle surface is the greatest. An effective way to increase the number of interfacial hydrogen bonds in nano-SiO_2_ and meta-aramid fibres is to increase the number of hydrogen atoms on the nano-silica surface and oxygen atoms in the meta-aramid fibre. By using the radial distribution function (RDF), the conclusion can be further drawn that the hydrogen bond formation probability is at a maximum when the atomic distance is 2.7–2.8 Å; therefore, increasing the number of atoms within this range can significantly increase the formation probability of hydrogen bonds. According to the results of chain movement, the existence of interfacial hydrogen bonds effectively limits the free movement of the molecular chains of meta-aramid fibres and enhances the thermal stability of meta-aramid fibres. The existence of interfacial hydrogen bonds is one of the important reasons for formation of the stable interface structure between nanoparticles and meta-aramid fibres. In addition, a nanoparticle with a small radius improves the interfacial hydrogen bond energy density and interfacial interaction energy density, enhancing the stability of the mixed model interface.

## 1. Introduction

Through the doping of nanoparticles for modified nano materials, the raw materials are provided with many new characteristics, and many properties of the raw materials are improved [1,2]. With the rapid development of nanotechnology, the application field of nano materials has been further expanded [3]. At present, there is a trend to incorporate nanoparticles in high polymer insulating materials to improve the properties of the materials, especially for the improvement of the voltage level in power systems, and this process requires further improvements in the insulating property, thermal stability, mechanical property, and so on, of the electrical insulating material [4]. As an important electrical insulating material, the aramid insulating paper will gradually be widely used [5,6,7,8], and on the study of the aspect of property improvement, modification by doping with nano-silica will gradually become a key research direction widely assumed by many scholars [9,10,11,12].

At present, the focus of the study of modified nanomaterials has gradually been transferred from the improvement of the macro-properties to the exploration of the microscopic mechanism, and, for example, in the documents of [13,14,15,16], and so on, the mechanism behind the improvement to the macro-properties by the nanoparticles is explained through an exploration of the microscopic mechanism. With the exploration of the microcosms, the microscopic mechanism behind the effect of nanomaterials on polymer properties is gradually revealed further. For example, in the research field of biotoxicology of nanomaterials, study of the interaction between nanomaterials and the interface of the biological system [17] is mainly focused on the dynamic process of physical and chemical interactions between the surfaces of nanomaterials, and the biological composition (such as protein, membrane, organelle, DNA, etc.) is included in the “nano-biology” interface; these processes include the exchange reaction kinetics and thermodynamic levels between the nanomaterials and various biomolecules in the biological system [18]. It is widely believed that the DNA double helix owes its stability to the hydrogen bonds holding the two strands together. However, in the dissociated state, the individual strands can form even stronger hydrogen bonds with the solvent water molecules [19]. Klaus Lackner’s group studied hydrogen-bond effects on the ions on the surface of polymer [20] and the hydrogen-bond can affect the interactions of different anions on polymers [21]. Qibin Li’s group found that the hydrogen bonds play an important role in the surface of hydrate [22,23]. The number of average hydrogen bonds per water molecule is little different in the mixture region and the hydrate region, close to the value of the saturation number of water molecules. The radial distribution function (RDF) of H_2_O particles and F3 order parameter indicate that the water is a crystal structure in the whole system [22]. The phase transition in the water/graphene-oxide system needs more time to break the network of hydrogen bonds among water molecules, thereby reducing the evaporation rate [23]. It can be seen that the hydrogen bond exists widely, and it has certain effects on the structure and properties of materials. Therefore, the hydrogen bond is an important non-negligible factor affecting the interface interactions [24]. 

The hydrogen bond is a weak force that widely exists between molecules or within the molecules and is similar to the electrostatic interaction. The hydrogen bond that forms between two molecules is called the intermolecular hydrogen bond. The hydrogen bond that forms between atoms in the same molecule is called the intramolecular hydrogen bond. A close relation [13] exists between the hydrogen bonding network of polymers and the melting point, boiling point, structure, cohesive energy density, and so on, of polymers, and a large effect [25] of hydrogen bonds on the mechanical properties and anti-aging properties of cellulose, and so on, has been considered. The intramolecular hydrogen bond in the body plays a pivotal role, for example, a close relation [26] exists between the helical structure of protein, activity of some biochemical molecules, and so on, and hydrogen bonding. In the preparation of macromolecular polymers, the purpose of changing the performance is achieved by mixing various polymers and changing the blend compositions. However, the number of discovered miscible polymer blends is small, and most polymers are not miscible with other polymers; their interface is clear, the bonding between compositions is not strong, and it can be seen in a study that the compatibility of immiscible copolymers can be strengthened by forming intermolecular hydrogen bonds to improve the properties of the polymer blend [27,28]. Furthermore, because a large number of hydroxide radicals exist on the surface of nano-SiO_2_, a coacervate with a dimensional network structure forms through hydrogen bond interactions among the nanoparticles. To decrease such an agglomeration phenomenon, the surface modification [3] of nanoparticles is carried out, and one of the purposes is to decrease the possibility of the occurrence of the agglomeration phenomenon by decreasing the number of hydrogen bonds at the nanoparticle interface. Xi Chen’s group studied different number of hydrogen bonds can affect the energy barriers of chemical reactions [29]. Hossein Eslami’s group found that: Addition of water molecules to PA-6,6 + water system, confined in wide pores, accumulates water molecules into clusters. In such wide pores, the amide groups are not as accessible to water molecules, therefore hydrogen bonding between the water polymer cannot stabilize the mixture [30]. Studies in the literature [31] show that, The number of hydrogen bonds per donor for free polymer chains decreases with increasing the grafting density, with a faster decrease for systems containing bigger nanoparticles compared to those with smaller nanoparticles. The maximum probability occurs at a distance about 0.15 nm from the surface. Further reports in the literature [32,33] show that: hydrogen-bond formation depends on the layering effect and on the geometrical restrictions and reveals an oscillatory behavior like the solvation force oscillations. The hydrogen bonding in PA-6,6 is responsible for the formation of a 3-D network, giving rise to the special mechanical properties of this polymer. Although the abovementioned results suggest the importance of interfacial hydrogen bonds and that the number of interfacial hydrogen bonds is an important factor affecting interaction, a further study on the important factors affecting the number of interatomic hydrogen bonds on the atomic scale has not been carried out, namely, a study on the formation probability of interatomic hydrogen bonding.

Additionally, the performance of nanocomposites is closely associated with the particle size and morphology of the materials, and the many properties of the composite, such as optical property, electricity property, magnetic property, and so on, can only be viewed at the macroscopic level when the particle size and shape of the materials are uniform [34,35]; thus, control of nanoparticle size has become a popular topic of research. For example, studies of the effects of nanoparticles with different sizes on the properties and the mechanisms behind the modified nanomaterials have been performed [36,37,38,39,40]. Although the studies carried out by many scholars at home and abroad show that nanoscale is one of the most important parameters for characterization of nanomaterials and that the effects of nanoparticles with different sizes on material properties and in the aspect of biological effects are different [41,42,43,44,45,46], further exploration is still required on the microcosmic mechanism behind the effect of the size of the nanoparticle on the properties of the modified materials.

On that account, this paper describes the doping modification of meta-aramid fibres by nano-silica, and studies the hydrogen bond interactions in the SiO_2_/meta-aramid fibre materials at the atomic scale. First, the pure meta-aramid fibre and mixed SiO_2_/meta-aramid fibre (with nanoparticle radiuses of 3, 5, 7 and 9 Å) models are built with the Materials Studio (MS) software, and then the optimization processing and dynamics simulation of the model are carried out. In this paper, the effect of the nanoparticle radius on the number of different types of hydrogen bonds is analysed by the statistics of the number of different types of hydrogen bonds in all the models. The formation probability of the hydrogen bonds among different atomic and SiO_2_/meta-aramid fibre interfaces is reflected through the pair correlation function and radial distribution function The effect of the nano-silica particle size on the thermal stability of the meta-aramid fibres is represented by using the mean square displacement (MSD) of meta-aramid fibres in different models. The effect of the interfacial hydrogen bond interactions on the overall interaction between the nanoparticle and meta-aramid fibre is described through changes in the interaction energy and hydrogen bond energy of the interface.

## 2. Formation, Model Building and Parameter Setting of Hydrogen Bonds

### 2.1. Formation of Hydrogen Bonds

The hydrogen bond generally refers to the bond formed through the certain bonding force generated between the H atom combined with a high-electronegativity element in a molecule and a high-electronegativity atom in another molecule. That is, D–H…A by formula, where D refers to the donor atom (donor), A refers to the acceptor atom (acceptor), and both of them represent atoms with great electronegativity and a small radius, such as F, O, N, and so on.

In D–H…A, D–H refers to the strong polar covalent bond, and because the electronegativity of D is great and the electronic attraction is strong, the hydrogen atom becomes a “naked” electron nearly without an electron cloud and with some positive charge. The radius of D is especially small, the electric field intensity of D is great, and no inner electron exists for D. The other atom, A (that is, the atom with great electronegativity, small radius and lone pair electrons), with some negative charge, is allowed to sufficiently reach D; therefore, a strong electrostatic interaction is generated, and the hydrogen bond is formed. Hydrogen bonding can be in the range of 2–8 kcal, and compared with the intermolecular action force (i.e., van der Waals force), the bonding is slightly strong; however, compared with the covalent bond and ionic bond, the bonding is weak. The hydrogen bond energy in the range of 25–40 kJ/mol belongs to a medium-strength hydrogen bond, and it is generally acknowledged that a bond is of weak hydrogen bond strength when the bond energy is less than 25 kJ/mol, whereas the bond is of a strong hydrogen bond when the bond energy is greater than 40 kJ/mol.

**Figure 1:**
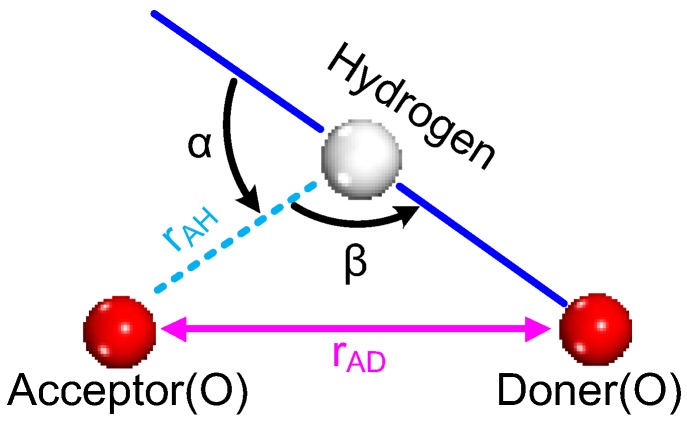
Schematic diagram for the definition of a hydrogen bond.

In classical molecular dynamics simulation, the definition of the hydrogen bond is vague [47,48]; therefore, two methods are usually adopted: energy criterion and the geometry rule. The definition of the hydrogen bond by the energy criterion is when the interaction energy between molecular pairs is greater than a certain value, and this interaction between the molecular pairs is deemed a hydrogen bond interaction [49]. The definition of hydrogen bond by the geometry rule is the relative position between two molecules [48,50,51], as shown in Figure 1; the maximum distance (*r*_AH_) between the acceptor atom and hydrogen atom is defined as 3 Å (angstrom, 1 Å = 10^−1^ nm), the minimum value of the included angle (β) is 90°, and the donor atom and acceptor atom are described using the oxygen atom as an example. In this paper, the definition of the hydrogen bond is made by the geometry rule [13].

### 2.2. Model Building

**Figure 2:**
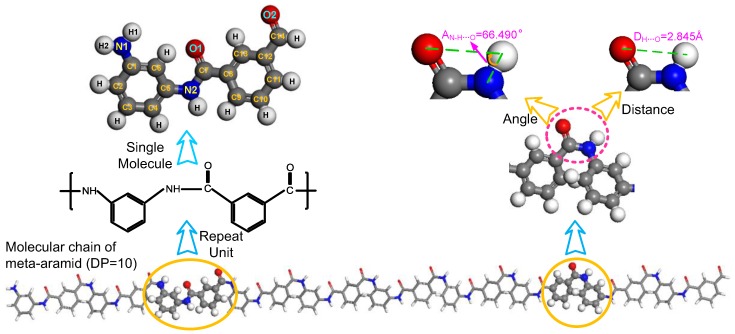
Meta-aramid fibre molecular model, polymerization degree (DP) = 10.

First, the meta-aramid fibre single-chain model is built, and the polymerization degree (DP) is 10, as shown in Figure 2. The left half of the figure indicates the repeating unit structure in the meta-aramid chain and a single meta-aramid molecular model, and the right half indicates the bond angle among atoms N, H and O and bond length of the atoms H and O.

**Figure 3:**
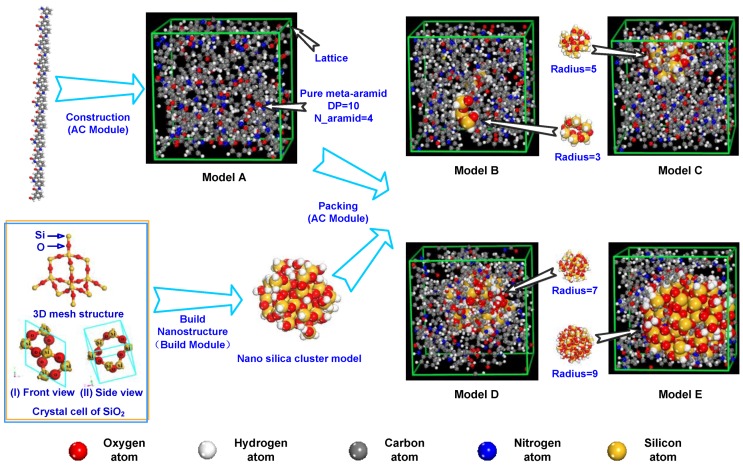
Model building process and built models; Model A is the pure meta-aramid fibre model, and Model B, Model C, Model D and Model E are the mixed models of the SiO_2_/meta-aramid fibre.

By using the Visualizer module in MS, an amorphous model can be built through the Amorphous Cell (AC) module, for example, Model A in Figure 3 is a pure meta-aramid fibre model. A periodic boundary condition is established. Then, the silica unit cell is imported to build the nano-silica cluster model. Because unsaturated residual bonds and hydroxide radicals with different bonds and status [52,53] exist on the surface of nano-SiO_2_, during the building of the nano-SiO_2_ cluster model, the position supplement is conducted with the hydrogen atom for the unsaturated chemical bond existing on the surface because of shearing. The mixed model of the SiO_2_/meta-aramid fibre is built with the Packing tool in the AC module. For the model building, the periodic boundary condition is established, the density is 1.2 g/cc, the chain number (N aramid) of the meta-aramid fibres is 4, the number of nano-SiO_2_ is 1, and the radiuses of nanoparticles are 3, 5, 7 and 9 Å. The figure for the building of the mixed models of nano-SiO_2_/meta-aramid fibre (Model B, Model C, Model D and Model E) is shown in Figure 3.

### 2.3. Simulation Parameter Setting

During the geometric optimization, annealing treatment and molecular dynamics simulation of the model, the COMPASS (condensed-phase optimized molecular potentials for atomistic simulation studies) force field applicable to the organic and inorganic molecules [44] is adopted as the force field. After the model is built, the energy is great and the model is unstable; therefore, a geometric optimization is carried out for all the models, and then an annealing treatment is conducted with the number of annealing cycles being 10, an initial temperature of 300 K, and the middle temperature of each cycle set as 900 K. After the complete annealing treatment, the model energy reaches a reasonable value, and the dynamics simulation is subsequently conducted. A 300 ps simulation for the model after relaxation is first carried out with the Discover module in the NVT (constant volume and temperature) ensemble, the simulation time step is 1 fs, the cut-off radius is set as 9.5 Å, and the spline width is set as 1 Å; subsequently, the NPT (constant pressure and temperature) ensemble is selected, the Andersen method is adopted as the temperature control method, the pressure intensity is set as the standard atmospheric pressure, and the information of the primary dynamics is collected with the Berendsen pressure controlling method every 50 steps.

## 3. Simulation Results and Discussion

### 3.1. Change in Number of Hydrogen Bonds in all the Models

**Figure 4:**
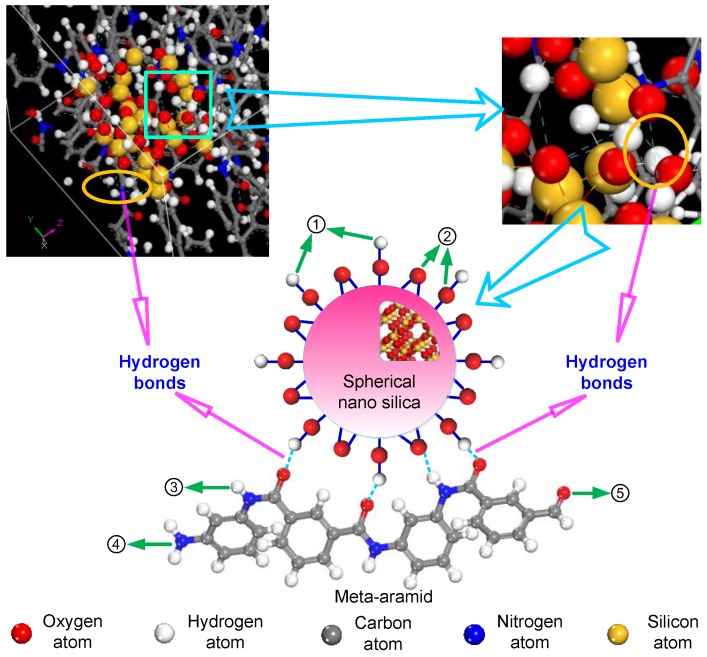
Hydrogen bonds formed between the nano-silica and meta-aramid fibre, ① indicates the hydrogen atom on the nano-silica surface, ② indicates the oxygen atom on the nano-silica surface, ③ indicates the hydrogen atom in the –NH radical in the meta-aramid fibre molecule, ④ indicates the nitrogen atom in the –NH radical in the meta-aramid fibre molecule, and ⑤ indicates the oxygen atom in the meta-aramid fibre molecule.

In the mixed model of the nano-SiO_2_/meta-aramid fibre, because the –NH radical and oxygen atom exist in the meta-aramid insulating paper fibre, the –NH…O and –NH…N hydrogen bonds are easily formed in the molecules and between the molecules of the meta-aramid fibre. –OH…O hydrogen bonds are easily formed on the surface of nano-silica. –OH…N, –OH…O and –NH…O hydrogen bonds are easily formed at the meta-aramid insulating paper fibre and silica nanoparticle interface. For a simplified description and instruction, the surficial hydrogen atom on the nano-silica, oxygen atom on the nano-silica, hydrogen atom in the meta-aramid fibre chain–NH radical, nitrogen atom in the meta-aramid fibre chain and oxygen atom in the fibre chain of the meta-aramid insulating paper are respectively marked as atoms ①, ②, ③, ④ and ⑤. Therefore, only ①…②, ①…④, ①…⑤, ②…③, ③…④ and ③…⑤ hydrogen bonds are formed in the model. Therein, ①…② hydrogen bonds are the hydrogen bonds on the nanoparticle; ③…④ and ③…⑤ hydrogen bonds are the hydrogen bonds in the meta-aramid fibre; and ①…④, ①…⑤ and ②…③ hydrogen bonds are the interfacial hydrogen bonds. The hydrogen bonds formed between the nano-silica and meta-aramid fibre are shown in Figure 4.

**Figure 5:**
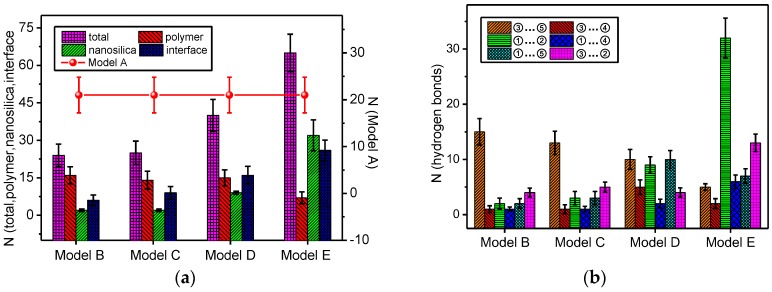
Number of hydrogen bonds, (**a**) indicates the number of different types of hydrogen bonds in all the models; and (**b**) indicates the number of hydrogen bonds between different atoms at the interface.

The number of hydrogen bonds in the model can be expressed as shown in the relational expression (1), where *N*_total_ means the total number of hydrogen bonds in the model, *N*_polymer_ means the number of meta-aramid fibre intramolecular and intermolecular hydrogen bonds, *N*_nanosilica_ means the total number of hydrogen bonds in nano-SiO_2_, and *N*_interface_ means the number of the hydrogen bonds between the nanoparticle and meta-aramid fibre molecule. The statistics of different types of hydrogen bonds in all the models after the dynamics simulation are shown in Figure 5.

(1) $$N_{\mathrm{total}}=N_{\mathrm{polymer}}+N_{\mathrm{nanosilica}}+N_{\mathrm{interface}}$$

It can be seen from Figure 5a that *N*_total_ of all the models doped with nanoparticles is greater than that of Model A, and this can indicate that doping with nanoparticles can beneficially increase the number of hydrogen bonds. With the increase in the radius of the nanoparticle, except for the total amount (*N*_polymer_) of intramolecular or intermolecular hydrogen bonds of meta-aramid fibres decreasing, the total number (*N*_total_) of the system hydrogen bonds, total number (*N*_nanosilica_) of the hydrogen bonds on the nano-silica particles and total number (*N*_interface_) of the nanoparticle and meta-aramid insulating fibre interfacial hydrogen bonds increase, and the rate of increase of these values follows the trend of the radius. The reasons for this change are as follows. On the one hand, in the model built in this paper, the number of surface hydroxide radicals increases sharply with the increase of nanoparticle radius, and this leads to an increase in the formation probability of hydrogen bonds on the nanoparticle and at the interface. On the other hand, in the dynamics process, because the distance and bond angle between atoms change due to the torsion and deformation of the molecular chains of the meta-aramid fibres, the fibre is readily found close to the nanoparticle, and the formation probability of hydrogen bonds is further increased, therefore, the *N*_nanosilica_ and *N*_interface_ values are increased. However, a decreasing trend is presented for the *N*_polymer_ value; this is mainly due to the fact that the greater the nanoparticle radius, the greater the volume of the mixed model, and the fewer contact opportunities for the atoms between the meta-aramid fibre chains, leading to the decrease of the formation probability of intramolecular or intermolecular hydrogen bonds in meta-aramid fibres. From the trend in the figure, when the nanoparticle radius is greater than 9 Å (Model E), the contribution of the *N*_nanosilica_ value to *N*_total_ value is the greatest; this is mainly due to the proportion of the nanoparticles gradually increasing, and the number of surface atoms and number of hydrogen bonds formed between the hydrogen and oxygen atoms on the nanoparticles increase with the increase of radius.

The change in the number of the different hydrogen bonds formed with the nanoparticle radius is shown in Figure 5b. In this figure, with the increase in radius, the increasing trend of nano-SiO_2_ intramolecular ①…② hydrogen bonds is evident, and when the radius is 7 Å, the nano-SiO_2_ bonds become the main contributor to the total number of hydrogen bonds. In the meta-aramid insulating paper fibre, a clear downtrend is presented for the number of ③…⑤ hydrogen bonds, while the number of ③…④ hydrogen bonds basically remains unchanged, and this indicates that a strong effect is generated on the ③…⑤ hydrogen bonds in the meta-aramid fibre from the dynamics motion limit. In the hydrogen bonds formed at the interface, the number of ①…⑤ hydrogen bonds keeps rising below the radius is 7 Å, and the number of hydroxide radicals on the nanoparticles increases sharply after the radius is 7 Å. The number of ①…⑤ hydrogen bonds shows a downward trend after the radius is 7 Å; the main reason for this is that the number of oxygen atoms contained in the meta-aramid fibres of the model remains the same and the motion of the meta-aramid fibre molecules are limited, which cause a decrease in the number of ①…⑤ hydrogen bonds instead of an increase when the radius is larger than 7 Å. Furthermore, in the hydrogen bonds formed at the interface, with the increase of radius, ②…③ hydrogen bonds and ①…④ hydrogen bonds basically show an upward trend, and ②…③ hydrogen bonds become the main contributor of the interfacial hydrogen bonds when the radius is greater than 7 Å. The number of ②…③ hydrogen bonds is always greater than the number of ①…④ hydrogen bonds, which indicates the bonding probability of the hydrogen atom bonded with the nitrogen atom in the meta-aramid fibre and the oxygen atom on the nano-silica molecule is greater than the bonding probability of the hydroxide radical on the nano-silica molecule and the N atom in the meta-aramid fibre.

### 3.2. Analysis of Formation Probability of Interatomic Hydrogen Bonds

#### 3.2.1. Pair Correlation Function

The PCF can be represented with the symbol *g*(*r*), and the distribution of the local space particles becomes an effective method for studying the interaction between the material structure and the particle [54]. This means that the conditional probability density that a particle β is found in the range of the radius from *r* to *r + dr,* where the particle *α* is centred.

**Figure 6:**
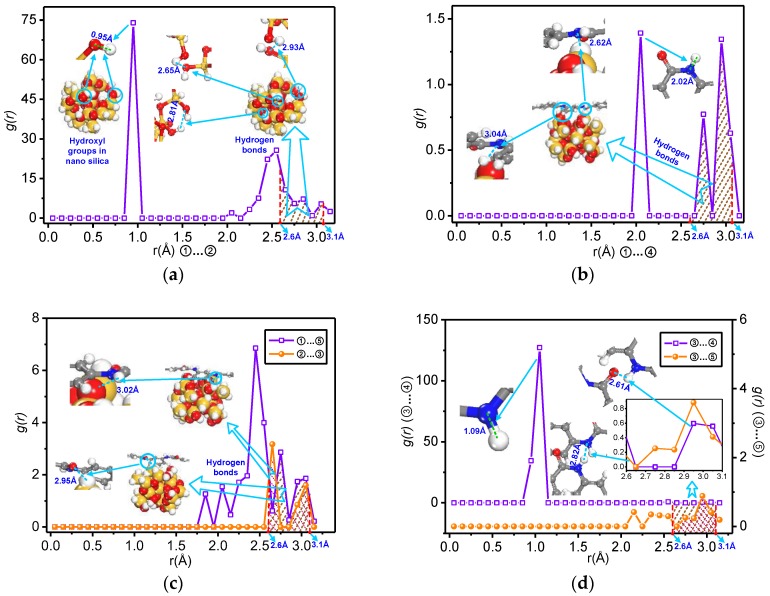
Pair correlation function for different types of hydrogen bond atoms of Model C at 90 °C: (**a**) ①…② hydrogen bonds; (**b**) ①…④ hydrogen bonds; (**c**) ①…⑤ and ②…③ hydrogen bonds; (**d**) ③…④ and ③…⑤ hydrogen bonds.

In this paper, the interaction condition between atoms and molecules is further obtained through the pair correlation function between these pairs of atoms. Here, Model C is taken as an example. The pair correlation functions for all the atoms are shown in Figure 6.

In Figure 6, the horizontal coordinate represents the distance between atoms, and the vertical ordinate represents the probability density [55]. The action range of hydrogen bonds is generally within 2.6–3.1 Å [54].

From Figure 6, when *r* = 0.95 Å, the first peak appears, similarly, in Figure 6d, when *r* = 1.05 Å and *g*(*r*) = 127.23, for the distance between atoms ③ and ④, a peak value appears. Because hydroxide radical exists in the nano-silica molecule and –NH bond exists in the meta-aramid fibre, the bond length of the oxygen atom and hydrogen atom in the hydroxide radical and the bond length of the nitrogen atom and hydrogen atom in the –NH bond are roughly equal to the distance (*r*) at the peak value. After a comparison of all the figures, it can be shown that the *g*(*r*) values are different with increasing radius, and this indicates that the occurrence probability of these atom pairs at the distance is different.

A further study is carried out through the hydrogen bond interactions of the model in the curve shown in Figure 6, where the statistics for certain hydrogen bond interactions is obtained by removing the curve with a radius of 2.6–3.1 Å. The calculation results are given by Formula (2), and if the formula is replaced with the indefinite integral of *g*_ij_(*r*), the integral will be the same as in KB (Kirkwood-Buff) theory [56]. The larger S is, the greater the hydrogen bond formation probability is, and P indicates the hydrogen bond formation probability. Because the minimum value of *g*(*r*) is 0, the minimum value of *S* is ‒0.5.

(2) $$S=\int_{2.6}^{3.1}\left[g(r)-1\right]dr$$

**Figure 7:**
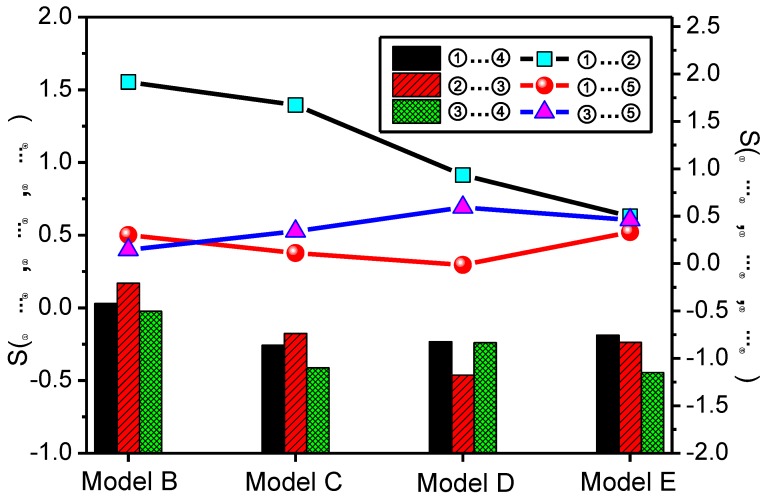
Relationship between all the atom pairs (*S*) and the radius of nano-silica at 90 °C.

The calculation results are as shown in Figure 7.

It can be seen in Figure 7 that decrease of *S*_①__…__②_ is great with increasing radius of the nano-silica, and *S*_②__…__③_ and *S*_①__…__④_ maintains a downtrend that is relatively gentle. For *S*_③__…__④_ in Model D, there is a slight increase, but the overall trend is downward; this indicates that the hydrogen bond formation probability of this atom pair decreases with the increase in the radius of the nanoparticle. *S*_①__…__⑤_ exhibits a downtrend at first, with the minimum value occurring when the radius is equal to 7 Å, then an uptrend occurs, and the change of *S*_③__…__⑤_ is opposite to that of *S*_①__…__⑤_. According to the changing relationships of the number of hydrogen bonds in the models, it can be seen that the number of hydrogen bonds is not positively related to the hydrogen bond formation probability of atom pairs, and the main reason is that the factors affecting the number of hydrogen bonds include the formation probability of a hydrogen bond and the number of atoms forming these hydrogen bonds. Although the formation probability of a hydrogen bond between atoms ① and ② decreases with the increase in radius, the number of hydroxide radicals and oxygen atoms on the nano-silica molecule surface increase sharply with the radius, and so this finally leads to an increase in the number of O–H…O (nanosilica) hydrogen bonds, with other atom pairs being similar.

*P* can be sorted in accordance with the value of *S*, taking Model C as an example, at 90 °C, the formation probability sequence of hydrogen bonds between the atom pairs in Model C is *P*_①__…__②_ > *P*_③__…__⑤_ > *P*_①__…__⑤_ > *P*_②__…__③_ > *P*_①__…__④_ > *P*_③__…__④__._

#### 3.2.2. Radial Distribution Function

There are three concepts of the radial distribution function, namely radial distribution functions of the wave function, the electron cloud and probability [57], and there are two definitions shown in Formulas (3) and (4) [58]. Formula (3) is adopted in this paper to express the radial distribution function [59], and the meaning of the formula is a statistical average of the atomic radial distribution in space.

(3) $$G(r)=r^{2}R^{2}(r)$$

(4) $$G(r)=4\pi r^{2}R^{2}(r)$$

The radial distribution function shows that if the particle α is deemed as the centre, the relationship of the probability of seeking a β particle in the range of the radius from *r* to *r + dr* with PCF is:

(5) $$g(r)=\frac{G(r)}{4\pi r^{2}\rho }$$

where

(6) $$\rho =\frac{N_{\beta }}{V}$$

*ρ* means the average density of particles, *N*_β_ means the total number of β particles, and *V* means the space volume. The PCF is a “normalization” process result of the RDF.

**Figure 8:**
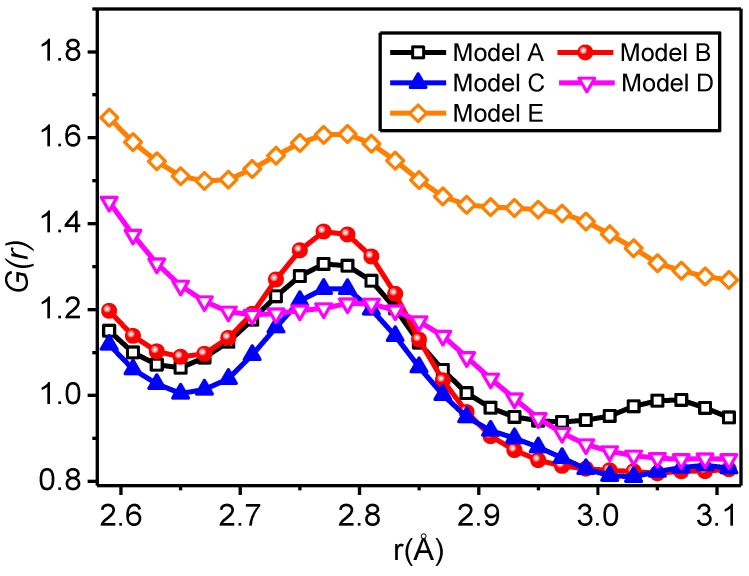
Total radial distribution functions (RDFs) of all models when *r* is within 2.6–3.1 Å.

In the RDF curve, the horizontal coordinate at the peak indicates the distance most likely to occur among atoms, the half width of the peak mainly shows the distribution of atomic spacing, and the peak area indicates the number of atoms distributed in the spacing, that is, the coordination number [60]. The distance range of hydrogen bonds is generally within 2.6–3.1 Å [54,55]; therefore, only total RDFs (atoms ①, ②, ③, ④ and ⑤) of all the models when r is within 2.6–3.1 Å are given, as shown in Figure 8.

A peak value of the model appears when r is within 2.75–2.80 Å, indicating that a hydrogen bond is most likely to appear at this position, and the occurrence probability of the hydrogen bonds of all the models in this position is different, with the sequence of Model E > Model B > Model A > Model C > Model D. When r is within 3.0–3.1 Å, the formation possibility of hydrogen bonds is small.

By analysing the formation probability of hydrogen bonds in atom pairs in Model C through PCF, the hydrogen bond formation probability sequence is obtained: *P*_①__…__②_ > *P*_③__…__⑤_ > *P*_①__…__⑤_ > *P*_②__…__③_ > *P*_①__…__④_ > *P*_③__…__④_. The results are consistent with the results drawn by using the number of hydrogen bonds. From the radial distribution function, the hydrogen bond formation probability in the case of the radiuses of 2.75–2.80 Å in all the models is the greatest, the possibility to form hydrogen bonds within 3.0–3.1 Å is the smallest, and to increase the number of hydrogen bonds, the number of atoms within a radius in the range of 2.75–2.8 Å should be increased as far as possible. In the interfacial hydrogen bond, the formation probability of ①…⑤ hydrogen bonds is greatest, that is, an effective way to increase the number of interfacial hydrogen bonds in nano-SiO_2_ and meta-aramid fibres is to increase the number of hydrogen atoms on the nano-silica surface and oxygen atoms in the meta-aramid fibre.

### 3.3. Thermal Stability of Meta-Aramid Fibres

From the MSD, the chain motion of the polymer can be obtained, and the greater the slope in the time curve, the fiercer the chain movement of the polymer and the weaker the thermal stability of the polymer. The relationship between the MSD and time [61] is represented by Formula (7); in the formula, ${\vec{r}}_{i}(t)$ represents the coordinates of the *i*-th atom at the moment of *t* in the system, and ${\vec{r}}_{i}(0)$ represents the initial coordinates of the atom.

(7) $$MSD=\langle {\left|{\vec{r}}_{i}(t)-{\vec{r}}_{i}(0)\right|}^{2}\rangle$$

**Figure 9:**
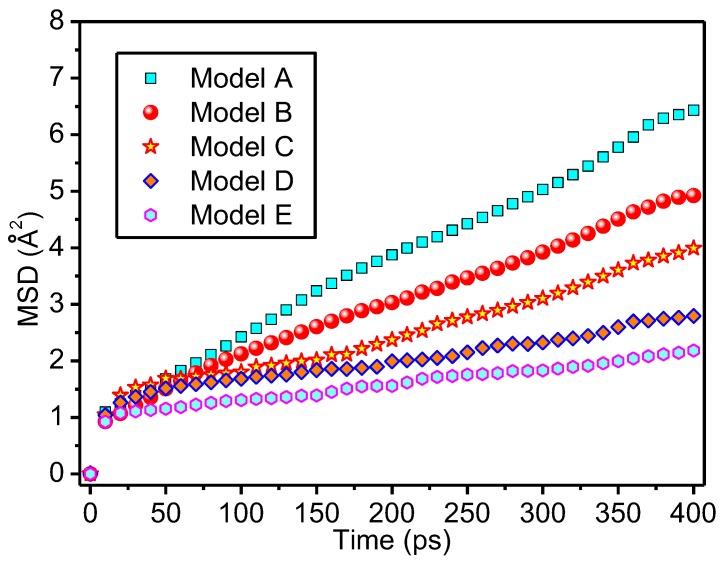
Mean square displacement of all the models.

The MSD of meta-aramid fibres in different models was shown in Figure 9. It indicates that the addition of nanoparticles reduces the strength of the meta-aramid fibres chain motion. For doping with nano-silica, a hydrogen bond is formed between the nanoparticle and the meta-aramid fibre, with binding for both caused by the interfacial hydrogen bond. The free movement of both is limited, thus, the chain movement of meta-aramid fibres is weakened. Therefore, compared with the unmodified model, the chain motion intensity of the modified model is clearly weakened. 

Moreover, the inhibition degree of nano-silica with different radiuses to the meta-aramid chain motion varies. In a certain case, this is mainly due to the contact area of the nanoparticle and meta-aramid increasing with the radius of the nano-silica particle; therefore, the number of the interfacial hydrogen bonds increases, and the binding effect on the meta-aramid fibre strengthens. Therefore, the larger the radius is, the weaker the chain movement of meta-aramid fibres is.

As the dynamics of hydrogen bonds are very fast and their length scales are very short, a more detailed analysis is needed to decide either the hydrogen bonds are responsible for the differences in MSDs as shown or there might be processed occurring at larger time and length scales (such as Coulombic interactions) which are mainly responsible for this. It might be that the effect of hydrogen bonds at the interface does not extend to such large time and length scales to affect the whole chain translation. The same phenomenon is addressed in the literature in [62], in charged systems capable of hydrogen bond formation. Therefore, the binding of interfacial hydrogen bonds to the free movement of meta-aramid fibres is limited and some more detailed analysis will be presented in a future paper.

### 3.4. Effects on Interaction Energy

The binding energy of the molecular polymer and nanoparticle surface greatly affects the properties of the high polymer and nanoparticle composites, and the impact degree often depends on the structural characteristics and microchemical properties of the high polymer and nanoparticle interface [63]. The intensity of the intermolecular interaction can be represented with intermolecular interaction energy, *E*_int_. If the value is less than 0, the molecular structure is steady, and the smaller the value is, the steadier the structural stability is. The interaction energy between the nanoparticle and meta-aramid is calculated with Formula (8).

(8) $$E_{\mathrm{int}}=E_{\mathrm{total}}-(E_{{\mathrm{SiO}}_{2}}+E_{\mathrm{ploymer}})$$

where *E*_total_ indicates the total potential energy of the meta-aramid and silica composite system, *E*_SiO2_ indicates the total potential energy of the nano-silica particle after the removal of the meta-aramid fibre, and *E*_polymer_ indicates the total potential energy of the meta-aramid fibre molecule chain after the removal of silica. *E*_total_ consists of the *E*_Valence energy_ value and *E*_Non-bond energy_ value, and the *E*_Non-bond energy_ value consists of *E*_Hydrogen bond energy_ value, *E*_van der Waals energy_ value, *E*_Electrostatic energy_ value, *E*_3-Body energy_ value and *E*_Restraint energy_ value. The energies of different models are shown in Table 1; therein, the *E*_H-interface_ indicates the bond energy of hydrogen bonds formed at the nanoparticle and meta-aramid interface.

As shown in Table 1, with the increase in the nanoparticle radius, *E*_int_ increases in the negative direction, the amplitude increases, and the trend of *E*_H-interface_ rises. This shows that *E*_H-interface_ provides a positive contribution to *E*_int_, and it indicates that the existence of interfacial hydrogen bonds is beneficial to the bonding of the two substances; that is, the interfacial hydrogen bond promotes the nano-silica particle to disperse in the meta-aramid fibres, to form a new type of insulating material. This shows that the existence of interfacial hydrogen bonds is one of the important reasons for the formation of the stable interface structure between nanoparticles and meta-aramid fibres. In spite of this, the hydrogen bond energy occupies a small proportion of the interaction energy; therefore, the effect of hydrogen bonds to the combination of both substances is still limited, and the effect of the degree of interfacial hydrogen bonds in different models on the interaction between the two substances is different. To analyse such a difference, the interfacial hydrogen bond energy density is utilized.

**Figure 10:**
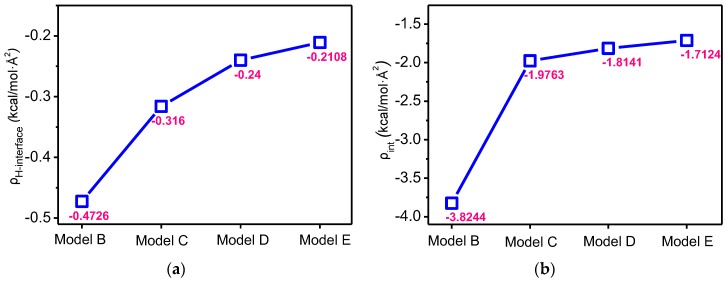
Change in bond energy density with the nanoparticle radius: (**a**) the change in hydrogen bond energy density with the radius of the nanoparticle and (**b**) the change in the interaction energy density with the nanoparticle radius.

The interfacial hydrogen bond energy density here is defined as the ratio of the interfacial hydrogen bond energy and the surface area of the nanoparticle, which is expressed as *ρ*_H-interface_. As given by Formula (9), where *E*_H-interface_ indicates the interfacial hydrogen bond energy, and *S*_nanoparticle_ indicates the surface area of the nanoparticle. Because the hydrogen bond energy is a negative value, the smaller the *ρ*_H-interface_ is, the stronger the effect of hydrogen bonds on the interaction between the two substances is. In the calculation, the circular constant is taken as 3.14, and the final results retain four decimal places as per the rounding-off method, as shown in Figure 10a.

(9) $$\rho_{H-\mathrm{interface}}=\frac{E_{H-\mathrm{interface}}}{S_{\mathrm{nanoparticle}}}$$

With the increase in radius, the *ρ*_H-interface_ gradually increases but the increase in amplitude is gradually reduced, which shows that with the increase of the nanoparticle radius, the effect of hydrogen bonds on the interaction between the two substances is weakened. Therefore, to improve the hydrogen bond interaction, a surface modification of the nanoparticle can be performed [64,65].

A similar function can be adopted to define the interfacial interaction energy density, *ρ*_int_, as shown in Formula (10). For the different models, the change in *ρ*_H-interface_ and *ρ*_int_ are shown in Figure 10b. The two functions have similar laws of change, and the results show that with the increase in the nanoparticle radius, the interaction per unit surface area weakens. Therefore, to improve *ρ*_int_ and *ρ*_H-interface_, when nano-silica is selected for the modification of meta-aramid fibres, nano-silica materials with small radiuses should be selected to the greatest extent.

(10) $$\rho_{\mathrm{int}}=\frac{E_{\mathrm{int}}}{S_{\mathrm{nanoparticle}}}$$

In summary, the existence of interfacial hydrogen bonds effectively restricts the free movement of meta-aramid fibres, reduces the chain movement of meta-aramid fibres, and increases the thermal stability of meta-aramid fibres. The interfacial hydrogen bond plays a critical role in the promotion of the bonding of the nano-silica particle and meta-aramid fibre. The interfacial hydrogen bond energy is an important constituent of the interaction energy, that is, the existence of interfacial hydrogen bonds promotes the interaction between the two substances, forming a relatively stable interface structure. Nanomaterials with small radiuses improve the interfacial hydrogen bond energy density and interface interaction energy density, enhancing the stability of the mixed model interface.

## 4. Conclusions

In this paper, the formation of hydrogen bonds between the nano-silica with different radiuses and the meta-aramid fibre interface is studied through molecular simulation. The hydrogen bond formation probability between atoms is analysed through the number of hydrogen bonds, pair correlation function and radial distribution function. The effect of the hydrogen bond on chain movement of meta-aramid fibres is analysed through the mean square displacement. The effect of the hydrogen bond on the interaction at the nano-silica and meta-aramid fibre interface is analysed by means of the interaction energy and hydrogen bond energy. The conclusions are as follows:

1. The total number of the hydrogen bonds of the mixed model increases with doping by nano-silica. With the increase of the nanoparticle radius, the number of hydrogen bonds on the nanoparticle surface and the mixed model interface of nano-SiO_2_/meta-aramid fibre increases; thus, the increasing trend of the –OH…O hydrogen bonds on the surface of nano-SiO_2_ is most evident.
2. For all of the hydrogen bond types of the SiO_2_/meta-aramid fibre mixed models, the hydrogen bond formation probability between the oxygen atom and hydrogen atom on the nanoparticle surface is the greatest. One of the effective ways to increase the hydrogen bonds in a SiO_2_/meta-aramid fibre is to increase the number of hydrogen atoms on the nano-silica surface and oxygen atoms in the meta-aramid fibre. Furthermore, to increase the formation probability of hydrogen bonds in all the models, the atomic distance should be enlarged to 2.7–2.8 Å as far as possible.
3. The existence of interfacial hydrogen bonds can restrict the free movement of meta-aramid fibres, lower the chain movement of meta-aramid fibres, and improve the thermal stability of fibres. Additionally, the existence of interfacial hydrogen bonds is one of the important reasons for the formation of the stable interface structure of nanoparticles and meta-aramid fibres. However, at larger time and length scales, some other influence factors (such as Coulombic interactions) which can act on the MSDs should be taken into consideration.

In the modification process of the meta-aramid fibre, although the increase of the nanoparticle radius results in an increase in the number of interfacial hydrogen bonds of the mixed model, generally, a nanoparticle with a small radius can preferably promote the interfacial hydrogen bond energy density and interface interaction energy density of the mixed model and then promote stability of the mixed model interface, facilitating a better nanoparticle doping effect.

## Figures and Tables

**Table 1:** Energies of different models (kcal/mol) (1 cal = 4.1868 J).

Model	*E*_int_	*E*_SiO2_	*E*_Polymer_	*E*_total_	*E*_H-interface_
B	−432.3091	−636.4831	−1171.2478	−2240.0400	−53.4254
C	−620.5648	−856.8470	−1476.5172	−2953.9290	−99.2254
D	−1116.4885	−5631.9281	−1316.9705	−8065.3871	−147.7210
E	−1742.1470	−13,262.3598	−1506.8031	−16,511.3099	−214.4546
